# Echocardiographic assessment of left cardiac structure and function in antiretroviral therapy (ART)‐naïve people living with HIV/AIDS

**DOI:** 10.1002/iid3.799

**Published:** 2023-04-12

**Authors:** Xing Hu, Yuan Zhang, Tong Zhang, Weihua Li, Jing Han, Xuhui Zhang, Fankun Meng

**Affiliations:** ^1^ Ultrasound and Functional Diagnosis Center, Beijing Youan Hospital Capital Medical University Beijing China; ^2^ Center for Infectious Disease, Beijing Youan Hospital Capital Medical University Beijing China; ^3^ Beijing Institute of Hepatology, Beijing Youan Hospital Capital Medical University Beijing China

**Keywords:** acquired immune deficiency syndrome, cardiac function echocardiography, human immunodeficiency virus, left ventricular diastolic dysfunction

## Abstract

**Background:**

Patients with human immunodeficiency virus (HIV) are at a significantly higher risk of cardiovascular disease (CVD) compared to HIV‐negative people. Left heart dysfunction is the most common cardiac complication in people living with HIV/acquired immune deficiency syndrome (PLWHA), and diastolic dysfunction is an important predictor of cardiovascular events. The aims of this study were (1) to detect changes in left cardiac structure and function in antiretroviral therapy (ART)‐naive PLWHA using echocardiography; and (2) to investigate the risk factors for the development of left ventricular diastolic dysfunction (LVDD) in ART‐naive PLWHA.

**Methods:**

We retrospectively included 105 ART‐naïve PLWHA and included 90 healthy subjects as controls to compare the differences in left heart structure and function between the two groups. Univariate and multifactorial logistic regression were employed to explore the risk factors of the development of LVDD in ART‐naive PLWHA.

**Results:**

The left ventricular end‐diastolic internal diameter (LVEDD), left ventricular mass index (LVMI), and left atrial volume index (LAVI) were significantly greater in PLWHA than in controls (*p* < .05). The E/A ratio, lateral e′ velocity, and mitral deceleration time were significantly lower in PLWHA than in controls (*p* < .05). Average E/e′ ratio was significantly higher in PLWHA than in controls (*p* < .05). Left ventricular ejection fraction (LVEF) and left ventricular fractional shortening (LVFS) were not significantly different between PLWHA and controls (*p* > .05). Multifactorial logistic regression analysis showed that age, body mass index (BMI), and CD4^+^ count <200 cells/μL were independent risk factors for LVDD in ART‐naive PLWHA (OR = 1.781, 1.228, 3.683, *p* < .05).

**Conclusions:**

Left ventricular systolic function did not differ between PLWHA and controls, and left ventricular diastolic function was lower in PLWHA than in controls. Age, BMI, and CD4^+^ count were independent factors affecting LVDD in ART‐naive PLWHA.

## Graphical abstract

Left ventricular systolic function did not differ between patients living with HIV/AIDS (PLWHA) and controls, and left ventricular diastolic function was lower in PLWHA than in controls. Age, body mass index, and CD4^+^ count were independent factors affecting left ventricular diastolic dysfunction in antiretroviral therapy‐naive PLWHA.

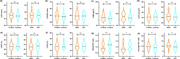

## 1 INTRODUCTION

Acquired immunodeficiency syndrome (AIDS) is a public health issue of global concern. Human immunodeficiency virus (HIV)‐specific invasion of CD4^+^ T lymphocytes leads to severe immune deficiency, which can cause damage to organs and tissues in various systems throughout the body. People living with HIV/AIDS (PLWHA) have a significantly higher risk of cardiovascular disease (CVD) than HIV‐negative people.^1^ CVD has become one of the leading causes of death from non‐AIDS‐related events in PLWHA, with its incidence exceeding that of other complications.^1^, ^2^ Chronic inflammation and persistent immune activation may be key mechanisms in PLWHA with co‐morbid CVD.^3^

Damage to the cardiovascular system in PLWHA is often subclinical, and its symptoms are easily masked by other comorbidities. Left heart dysfunction is the most common cardiac complication in PLWHA, and diastolic dysfunction is an important predictor of cardiovascular events.^4^, ^5^ Timely and accurate assessment of the cardiac function status in PLWHA is important to guide the clinical treatment and improve the management of PLWHA. Previous studies have mostly focused on cardiac involvement in PLWHA after long‐term antiretroviral therapy (ART),^5^ and some studies have shown that a risk of CVD in PLWHA is associated with ART, such as the use of protease inhibitors (PI).^6^, ^7^ There is limited research in patients with primary HIV infection who are not yet received ART. It is unclear whether HIV infection itself and its associated factors affect cardiac structure and function. To observe the isolated effects of HIV on cardiac dysfunction, our study uniquely captures participants during a time period before ART‐initiation, which isolates the effects of ART on study outcomes.

The aims of this study were (1) to detect changes in left cardiac structure and function in ART‐naive PLWHA using echocardiography and (2) to investigate the risk factors for the development of left ventricular diastolic dysfunction (LVDD) in ART‐naive PLWHA. This research has implications for managing CVD in PLWHA.

## 2 METHODS

### 2.1 Population

This was a cross‐sectional observational study in which we included patients initially confirmed as HIV‐positive diagnosis who attended the AIDS Clinic at Beijing Youan Hospital, Capital Medical University, from January 2021 to October 2022. Inclusion criteria were: (1) HIV infection confirmed by both initial screening and confirmatory tests, (2) no initiation of ART, (3) age of 18–60 years, and (4) available clinical and laboratory data. Exclusion criteria were: (1) comorbid congenital or acquired cardiac disease, (2) comorbid cardiac arrhythmias, (3) comorbid respiratory disease, (4) comorbid chronic kidney disease, (5) comorbid severe HIV‐related complications such as malignant lymphoma, pneumocystis carinii pneumonia, cryptococcal meningitis, etc. and (6) breastfeeding or pregnant women. PLWHA were divided into two groups according to CD4 count: AIDS group (CD4 count < 200 cells/μL) and HIV+ group (CD4 count ≥ 200 cells/μL). Age‐ and sex‐matched HIV‐uninfected individuals who underwent physical examination during the same period were included as controls. The controls had no clinical evidence of tumor, inflammatory disease, CVD, and metabolic disease by history, physical examination, and laboratory testing. The study was approved by the Research Ethics Committee of Beijing Youan Hospital, Capital Medical University (Protocol No. LL‐2020‐084‐K), and informed consent was obtained from all participants.

### 2.2 Clinical evaluation

General information, including age, sex, body mass index (BMI), disease history, HIV exposure, and duration of HIV was collected from the subjects. We retrieved laboratory data including total cholesterol (TC), triglycerides (TG), low‐density lipoprotein cholesterol (LDL‐C), high‐density lipoprotein cholesterol (HDL‐C), CD4^+^ T lymphocyte count, CD4^+^/CD8^+^ ratio, and viral load within 1 week of echocardiography.

### 2.3 Echocardiography

Complete transthoracic echocardiography was performed for all subjects using the ACUSON SC2000 ultrasound scanner (4V1c probe). Image acquisition and measurements were performed by two experienced sonographers in accordance with the latest guidelines recommended by the American Society of Echocardiography.^8^ Left atrial diameter (LAD), left ventricular end‐diastolic diameter (LVEDD), left ventricular end‐systolic diameter (LVESD), interventricular septum thickness (IVST), and left ventricular posterior wall thickness (LVPWT) were measured under the parasternal long‐axis view. Biplane Simpson's method was used to measure left ventricular ejection fraction (LVEF) and left ventricular fractional shortening (LVFS), a normal LVEF was >55%. In the apical four‐chamber view, the pulsed‐wave Doppler technique was used to measure peak early‐diastolic flow velocity (E), peak end‐diastolic flow velocity (A), and E‐wave deceleration time (DT) to calculate the E/A ratio of the mitral valve; tissue Doppler imaging was employed to measure early‐diastolic mitral annular velocity and the mean e’ was obtained by calculate the average of septal e′ velocity and lateral e′ velocity, then calculate the ratio of E to the mean e′ (E/e′) in early diastole.

At the end of ventricular systole, the endocardium of the left atrial wall was tracedin the apical four‐ and two‐chamber views, respectively, and the left atrial volume (LAV) was obtained using the long ellipsoid method.^9^ The left atrial volume index (LAVI) was calculated by dividing LAV by body surface area (BSA). Left ventricular mass (LVM) was calculated according to the formula recommended by Devereux et al.^9^: LVM (g) = 0.8 × [1.04 × (IVST + LVPWT + LVEDD)^3^‐LVEDD^3^] + 0.6(g), and the left ventricular mass index (LVMI) was obtained by dividing LVM by BSA. Left ventricular end diastolic volume (LVEDV) was assessed using the modified Simpson's rule, and left ventricular end diastolic volume index (LVEDI) was calculated by dividing LVEDV by BSA.

We assessed left ventricular diastolic function and graded it according to the 2016 ASE/EACVI guidelines.^10^ When sinus rhythm and LVEF were normal, at least three of the following conditions needed to be met to suggest LVDD: (1) mean E/e′ ratio > 14, (2) septal e′ < 7 cm/s and/or lateral e′ < 10 cm/s, (3) Tricuspid regurgitation peak velocity (TR_Peak)_ > 2.8 m/s, and (4) LAVI > 34 mL/m^2^. When mitral E/A ratio ≤ 0.8, E ≤ 50 cm/s, and two or three of the three criteria were negative (mean E/e′ratio > 14, TR_Peak_ > 2.8 m/s, LAVI > 34 mL/m^2^), it indicated grade 1 diastolic dysfunction; when mitral E/A ratio ≤ 0.8 and E > 50 cm/s, or if the mitral E/A ratio was > 0.8 but < 2, and more than two of the three criteria were positive, it suggested grade 2 diastolic dysfunction; when mitral E/A ratio ≥ 2, it was diagnosed as grade 3 diastolic dysfunction.

### 2.4 Statistical analysis

Statistical analyses were performed using SPSS 25.0 software. Normality and variance homogeneity tests were carried out for quantitative data and those with normal distribution are expressed as mean ± standard deviation ($\bar{x}$ ± s), intergroup comparisons were conducted using independent‐samples *t*‐test; and those with nonnormal distribution are expressed as median (*P*25‐*P*75), intergroup comparisons were carried out using the Mann–Whitney *U* test. Categorical data are expressed as number (percentage), and the *χ* ^2^ test was used for intergroup comparisons. Risk factors for LVDD in ART‐naive PLWH were analyzed using univariate logistic regression, and variables with *p* < .1 in the univariate analysis were included in the multivariate logistic regression model. Pearson's correlation analysis was performed to assess the correlation between ultrasound parameters and CD4 cell counts. A two‐sided test was used, and a difference with *p* < .05 was considered to be statistically significant.

## 3 RESULTS

### 3.1 Population

The basic characteristics of the enrolled ART‐naive PLWHA and controls are detailed in Table 1. A total of 105 PLWHA were enrolled, with a mean age of 38.7 ± 9.2 years, including 92 men. There were 37 PLWHA with CD4 count < 200 cells/μL (AIDS group) and 68 PLWHA with CD4 count ≥ 200 cells/μL (HIV + group). The control group comprised 90 subjects with a mean age of 38.0 ± 9.2 years, including 71 men. There were no significant differences in age, sex, BMI, hypertension, diabetes, or smoking history between PLWHA and controls (*p* > .05). TC and HDL‐C were significantly lower and TG was significantly higher in PLWHA than in controls (*p* < .05). LDL‐C was not significantly different between the two groups (*p* > .05).

**Table 1:** Demographic and clinical characteristics of PLWHA and controls.

	PLWH (*n* = 105)	Controls (*n* = 90)	*p*‐Value
Age (years)	38.7 ± 9.2	38.0 ± 9.2	.585
Male (*n*, %)	92 (87.6%)	71 (78.9%)	.101
BMI (kg/m^2^)	22.8 ± 3.3	23.2 ± 2.7	.452
Hypertension (*n*, %)	12 (11.4%)	11 (12.2%)	.864
Diabetes (*n*, %)	13 (12.4%)	9 (11.1%)	.784
Smoking history (*n*, %)	33 (31.4%)	34 (37.8%)	.352
HIV exposure			
Sexual (*n*, %)	86 (81.9%)	‐	‐
Blood (*n*, %)	11 (10.5%)	‐	‐
IVDU (*n*, %)	8 (7.6%)	‐	‐
Duration of HIV infection (months)	6 (4–8)		
TC (mmol/L)	4.21 (3.78–4.70)	4.60 (4.15–5.00)	.003*
TG (mmol/L)	1.43 (1.04–1.95)	1.30 (1.00–1.50)	.016*
LDL‐C (mmol/L)	2.51 ± 0.68	2.65 ± 0.58	.130
HDL‐C (mmol/L)	1.13 ± 0.35	1.25 ± 0.31	.013*
CD4^+^ Count (cells/μL)	231 (130–387)	‐	‐
CD4^+^/CD8^+^ Ratio	0.55 (0.34–0.78)	‐	‐
viral load (copies/mL)	16,100 (494–50667)	‐	‐

*Note*: *p < .05.

Abbreviations: BMI, body mass index; HDL‐C, high‐density lipoprotein cholesterol; HIV, human immunodeficiency virus; IVDU, intravenous drug users; LDL‐C, low‐density lipoprotein cholesterol; PLWHA, patients living with HIV/AIDS; TC, total cholesterol; TG, triglycerides.

### 3.2 Comparison of the left heart structures and function between PLWHA and controls

The LVEDD, LVMI, LVEDV, LVEDI, and LAVI were significantly greater in PLWHA than in controls (*p* < .05). There were no significant differences in IVST, LVPWT, LAD, and LVESD between PLWHA and controls (*p* > .05) (see Table 2). The E/A ratio, lateral e′ velocity, and DT were significantly lower in PLWHA than in controls (*p* < .05). Average E/e′ ratio was significantly higher in PLWHA than in controls (*p* < .05). There were no significant differences in LVEF, LVFS, and Septal e′ velocity between the two groups (*p* > .05) (see Table 2).

**Table 2:** Comparison of left heart structure and function between PLWHA and controls.

		PLWHA (*n* = 105)	Controls (*n* = 90)	*p*‐Value
Cardiac structure	IVST (mm)	9.0 (7.5–10.0)	8.0 (7.0–9.0)	.070
	LVPWT (mm)	9.0 (8.0–10.0)	9.0 (8.0–10.0)	.431
	LAD (mm)	35.08 ± 3.62	34.42 ± 3.64	.212
	LVEDD (mm)	48.53 ± 4.47	46.25 ± 3.45	.026*
	LVESD (mm)	29.32 ± 3.68	28.50 ± 3.06	.094
	LVMI (g/m^2^)	83 (75–94)	79 (73–85)	.007**
	LAVI (mL/m^2^)	32.33 ± 5.93	30.67 ± 5.01	.037*
	LVEDV (mL)	110 (92–123)	103 (86–115)	.020*
	LVEDI (mL/m^2^)	65 ± 12	61 ± 9	.017*
Systolic function	LVEF (%)	66.34 ± 5.68	67.64 ± 5.31	.102
	LVFS (%)	37.02 ± 4.82	37.74 ± 4.53	.283
	Mitral E/A ratio	0.99 ± 0.28	1.13 ± 0.30	.001**
	Septal e′ velocity (cm/s)	8.14 ± 1.99	8.61 ± 1.97	.103
	Lateral e′ velocity(cm/s)	10.29 ± 1.37	11.21 ± 2.62	.002**
	Average E/e′ ratio	9.30 ± 2.70	8.36 ± 3.21	.028*
	Mitral DT (ms)	203 (189–222)	221 (200–231)	.001**

*Note*: *p < .05; **p < .01.

Abbreviations: DT, deceleration time; E/A, ratio of early‐diastolic LV inflow velocity (E) to atrial‐systolic velocity (A); E/e′, ratio of early‐diastolic LV inflow velocity (E) to early‐diastolic mitral annular velocity (e′); e’, early‐diastolic mitral annular velocity; IVST, interventricular septum thickness; LAD, left atrium diameter; LAVI, left atrial volume index; LVEDD, left ventricular end‐diastolic diameter; LVEDI, left ventricular end diastolic volume index; LVEDV, left ventricular end diastolic volume; LVEF, left ventricular ejection fraction; LVESD, left ventricular end‐systolic diameter; LVFS, left ventricular fractional shortening; LVMI, left ventricular mass index; LVPW, left ventricular posterior wall thickness; PLWHA, patients living with HIV/AIDS.

We assessed the left ventricular diastolic function of subjects according to the 2016 ASE/EACVI guidelines^10^: 62 of the 105 (59.0%) enrolled PLWHA had LVDD, and 20 of the 90 (22.2%) controls had LVDD. The prevalence of LVDD was significantly higher in PLWHA than in controls (*p* < .05). Of the 62 PLWHA with LVDD, grade 1 diastolic dysfunction was detected in 39 PLWHA (62.9%), grade 2 diastolic dysfunction was detected in 20 PLWHA (32.3%), and only three PLWHA (4.8%) had grade 3 diastolic dysfunction. Two of the 105 (1.9%) PLWHA had abnormal cardiac systolic function. The details of the two patients who had LV systolic dysfunction are shown in Supporting Information: Table S1.

### 3.3 Comparison of left heart structure and function between AIDS and HIV+ groups

**Figure 1:**
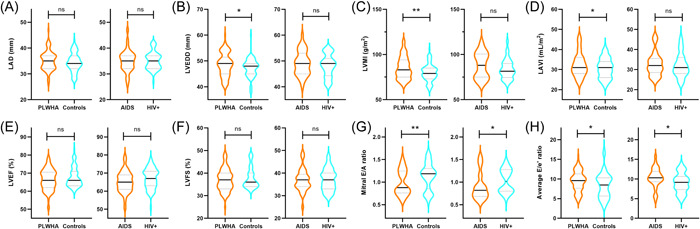
Violin plot showed differences in cardiac parameters between patients living with HIV/AIDS (PLWHA) and controls, and AIDS and HIV+ groups, *p < .05; **p < .01; ns: no significant difference; AIDS, acquired immune deficiency syndrome; HIV, human immunodeficiency virus; (A) LAD, left atrium diameter; (B) LVEDD, left ventricular end‐diastolic diameter; (C) LVMI, left ventricular mass index; (D) LAVI, left atrial volume index; (E) LVEF, left ventricular ejection fraction; (F) LVFS, left ventricular fractional shortening; (G) Mitral E/A ratio of early‐diastolic LV inflow velocity (E) to atrial‐systolic velocity (A); (H) Average E/e′ ratio of early‐diastolic LV inflow velocity (E) to early‐diastolic mitral annular velocity (e′).

The E/A ratio was significantly lower, the E/e′ ratio and LVEDI was significantly higher in AIDS group than in HIV+ group (*p* < .05). There were no significant differences in other left heart structural and functional parameters between AIDS and HIV+ group (*p* > .05) (see Table 3, Figure 1, and Supporting Information: Figure S1).

**Table 3:** Comparison of left heart structure and function between AIDS and HIV+ groups.

		AIDS (*n* = 37)	HIV+ (*n* = 68)	*p*‐Value
Cardiac structure	IVST (mm)	9.0 (8.0–10.0)	8.5 (7.0–9.8)	.173
	LVPWT (mm)	10.0 (9.0–10.0)	9.0 (8.0–10.0)	.252
	LAD (mm)	35.05 ± 4.12	35.09 ± 3.35	.963
	LVEDD (mm)	49.14 ± 4.70	48.21 ± 4.35	.312
	LVESD (mm)	28.86 ± 4.06	29.57 ± 3.46	.348
	LVMI (g/m^2^)	88 (75–101)	82 (75–90)	.189
	LAVI (mL/m^2^)	32.76 ± 5.94	32.10 ± 5.96	.592
	LVEDV (mL)	114 (105‐127)	108 (86‐123)	.053
	LVEDI (mL/m^2^)	69 ± 12	62 ± 11	.007**
Systolic function	LVEF (%)	65.16 ± 5.80	67.00 ± 5.55	.117
	LVFS (%)	36.95 ± 4.96	37.06 ± 4.79	.909
	Mitral E/A ratio	0.90 ± 0.30	1.03 ± 0.26	.027*
	Septal e′ velocity (cm/s)	7.91 ± 2.06	8.26 ± 1.96	.391
	Lateral e′ velocity(cm/s)	9.95 ± 1.28	10.48 ± 1.39	.057
	Average E/e′ ratio	10.32 ± 2.48	9.02 ± 2.49	.012*
	Mitral DT (ms)	194 (187–224)	206 (189–220)	.589

*Note*: *p < .05; **p < .01.

Abbreviations: AIDS, acquired immune deficiency syndrome; DT, deceleration time; E/A, ratio of early‐diastolic LV inflow velocity (E) to atrial‐systolic velocity (A); E/e′, ratio of early‐diastolic LV inflow velocity (E) to early‐diastolic mitral annular velocity (e′); e′, early‐diastolic mitral annular velocity; HIV, human immunodeficiency virus; IVST, interventricular septum thickness; LAD, left atrium diameter; LAVI, left atrial volume index; LVEDD, left ventricular end‐diastolic diameter; LVEDI, left ventricular end diastolic volume index; LVEDV, left ventricular end diastolic volume; LVEF, left ventricular ejection fraction; LVESD, left ventricular end‐systolic diameter; LVFS, left ventricular fractional shortening; LVMI, left ventricular mass index; LVPW, left ventricular posterior wall thickness.

### 3.4 Correlation of the left heart structural and functional parameters with CD4 cells in PLWHA

**Figure 2:**
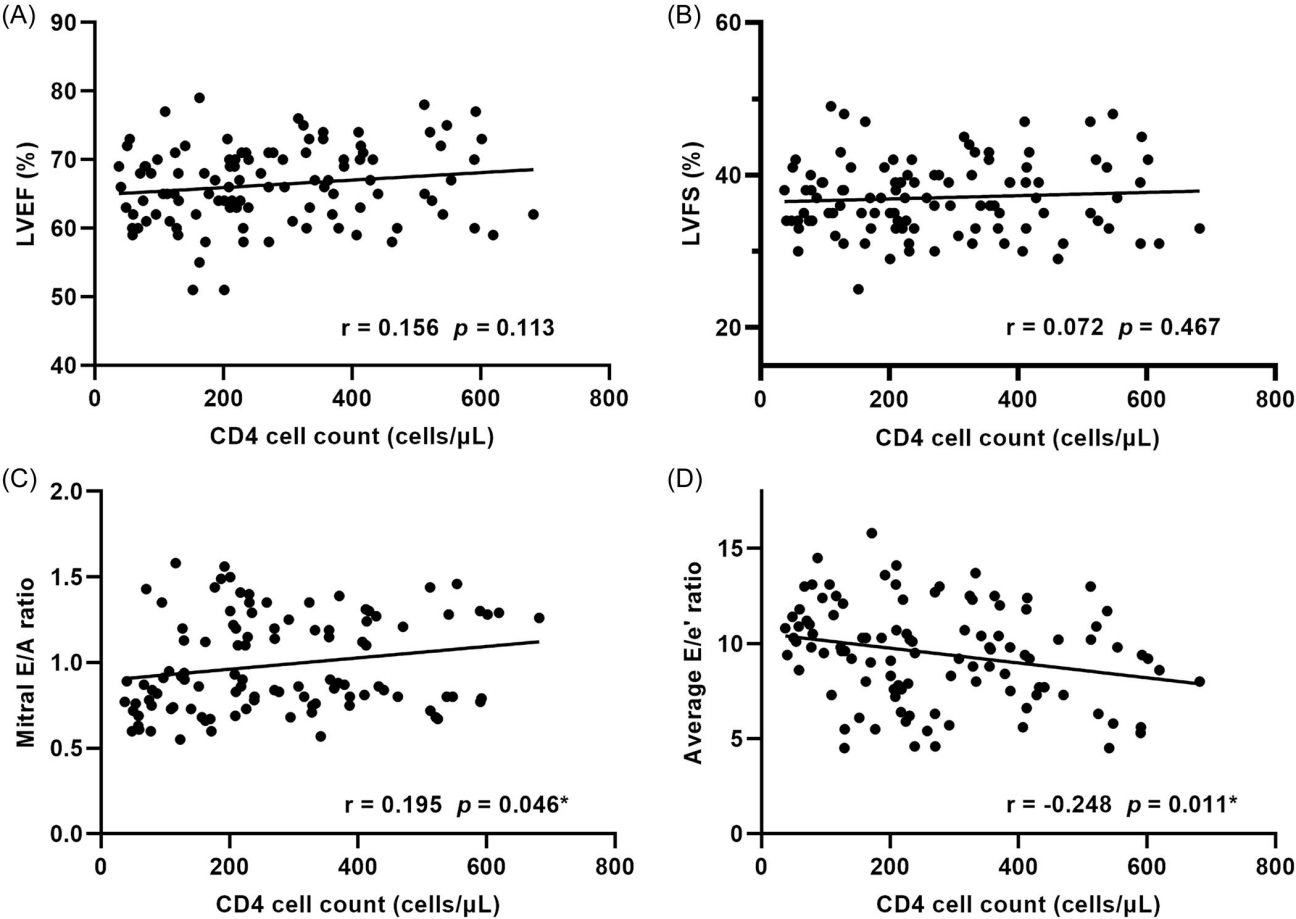
Scatter plots showed the correlation between CD4 cell count and (A) LVEF; (B) LVFS; (C) Mitral E/A ratio; (D) Average E/e′ ratio in PLWHA, *p < .05; Pearson's correlation analysis was performed to assess the correlation between ultrasound parameters and CD4 cell counts. PLWHA, patients living with HIV/AIDS; LVEF, left ventricular ejection fraction; LVFS, left ventricular fractional shortening; E/A, ratio of early‐diastolic LV inflow velocity (E) to atrial‐systolic velocity (A); E/e′, ratio of early‐diastolic LV inflow velocity (E) to early‐diastolic mitral annular velocity (e′).

The correlation between left heart structural and functional parameters of PLWHA and CD4 cell count was analyzed, as detailed in Table 4, Figure 2, and Supporting Information: Figure S2. The E/A ratio was positively correlated with CD4 cell count, the E/e′ ratio and LVEDI was negatively correlated with CD4 cell count, with correlation coefficients of 0.195 and −0.248, respectively (*p* < .05). There was no clear correlation between other cardiac ultrasound parameters and CD4 cell count (*p* > .05).

**Table 4:** Correlation of the left heart structural and functional parameters with CD4 cells count in PLWHA.

	Pearson's correlation coefficient (*r*)	*p*‐Value
IVST (mm)	−.102	.298
LVPWT (mm)	−.154	.116
LAD (mm)	−.036	.719
LVEDD (mm)	−.114	.247
LVESD (mm)	.083	.399
LVMI (g/m^2^)	−.029	.767
LAVI (mL/m^2^)	−.071	.471
LVEDV (mL)	−.176	.072
LVEDI (mL/m^2^)	−.253	.009
LVEF (%)	.156	.113
LVFS (%)	.072	.467
Mitral E/A ratio	.195	.046*
Septal e′ velocity (cm/s)	.009	.925
Lateral e′ velocity(cm/s)	.102	.298
Average E/e′ ratio	−.248	.011*
Mitral DT (ms)	.103	.296

*Note*: *p < .05. Pearson's correlation analysis was performed to assess the correlation between ultrasound parameters and CD4 cell counts. Abbreviations: AIDS, acquired immune deficiency syndrome; DT, deceleration time; E/A, ratio of early‐diastolic LV inflow velocity (E) to atrial‐systolic velocity (A); E/e′, ratio of early‐diastolic LV inflow velocity (E) to early‐diastolic mitral annular velocity (e′); e′, early‐diastolic mitral annular velocity; HIV, human immunodeficiency virus; IVST, interventricular septum thickness; LAD, left atrium diameter; LAVI, left atrial volume index; LVEDD, left ventricular end‐diastolic diameter; LVEDI, left ventricular end diastolic volume index; LVEDV, left ventricular end diastolic volume; LVEF, left ventricular ejection fraction; LVESD, left ventricular end‐systolic diameter; LVFS, left ventricular fractional shortening; LVMI, left ventricular mass index; LVPW, left ventricular posterior wall thickness.

### 3.5 Logistic regression analysis of risk factors for LVDD in PLWHA

To investigate the factors influencing LVDD in ART‐naive PLWHA, we performed a univariate regression analysis with the following factors as independent variables: age (per 10‐year increase), BMI, hypertension, diabetes, smoking history, duration of HIV infection, TC, TG, HDL‐C, LDL‐C, viral load (log‐transformed), and CD4 count. Variables with *p* < .1 in the univariate analysis, including age, BMI, hypertension, TG, HDL‐C, and CD4 count, were included in the multivariate regression analysis, which finally showed that age, BMI, and CD4 count were independent influencing factors of LVDD in ART‐naive PLWHA, see Table 5. Increased CD4 cell count is a protective factor for left ventricular diastolic function in ART‐naive PLWHA.

**Table 5:** Logistic regression analysis of risk factors associated with LVDD in PLWHA.

Variable	Univariate		Multivariate	
	Odds ratio (OR) (95% confidence interval [CI])	*p*‐Value	OR (95% CI)	*p*‐Value
Age (per 10‐year increase)	1.839 (1.186–2.849)	.006*	1.781 (1.076–2.947)	.025*
Body mass index	1.249 (1.058–1.475)	.009*	1.228 (1.016–1.485)	.033*
Hypertension	3.537 (0.942–13.282)	.061**	3.427 (0.805–14.583)	.096
Diabetes	1.697 (0.413–6.968)	.463	‐	‐
Smoking history	1.323 (0.566–3.092)	.518	‐	‐
Duration of HIV infection (months)	1.056 (0.963–1.159)	.244	‐	‐
TC (mmol/L)	1.135 (0.771–1.672)	.522	‐	‐
TG (mmol/L)	1.451 (0.948–2.220)	.087**	1.067 (0.616–1.847)	.817
LDL (mmol/L)	1.517 (0.835–2.757)	.172	‐	‐
HDL (mmol/L)	0.203 (0.056–0.739)	.016*	0.263 (0.048–1.441)	.124
Viral load (log‐transformed)	1.202 (0.854–1.692)	.291	‐	‐
CD4^+^ Count 200 cells/μL	3.111 (1.279–7.567)	.012*	3.683 (1.357–9.995)	.010*

*Note*: *p < .05; **p < .01. Variables with *p* < .1 in the univariate analysis were included in the multivariate logistic regression model.

Abbreviations: HDL‐C, high‐density lipoprotein cholesterol; LDL‐C, low‐density lipoprotein cholesterol; LVDD, left ventricular diastolic dysfunction; PLWHA, patients living with HIV/AIDS; TC, total cholesterol; TG, triglycerides.

## 4 DISCUSSION

Our study showed slightly increased changes in left ventricular internal diameter in ART‐naive PLWHA, suggesting that HIV can cause initial changes in the cardiac structure, including an increase in LVM.^11^ Increased LVM is associated with increased all‐cause mortality in both individuals without HIV infection and children with AIDS.^12^, ^13^ Our findings also suggested that the rate of abnormal left ventricular diastolic function was significantly higher in ART‐naive PLWHA than in the general population and that the occurrence of LVDD was independently associated with age, BMI, and CD4^+^ T lymphocyte count.

HIV infection can lead to dyslipidemia, and our study showed that ART‐naïve PLWHA had significantly lower HDL‐C and TC and higher TG levels, consistent with the results reported in previous papers.^14^, ^15^ The redistribution of visceral adipose tissue after HIV infection, and the development of dyslipidemia are associated with peripheral fat loss and trunk fat accumulation.^16^, ^17^ HIV infection is associated with disturbed lipid metabolism, leading first to an early decline in TC and finally to an increase in TG.^18^ The mechanism responsible for the early decrease in TC is unclear and may be associated with an increased predominance of small LDL particles.^19^ Persistent viral replication induces inflammation and secretion of cytokines such as interferon‐alpha (IFN‑α), which is thought to contribute to elevated TG levels.^20^ Low HDL levels may be associated with the secretion of soluble trans‐activator protein (TAT) by HIV in plasma, leading to reduced hepatocyte cholesterol mobilization.^21^ HDL‐C transports cholesterol from the vascular wall to the liver for catabolism and exerts an anti‐atherosclerotic effect. HDL‐C levels in HIV‐infected patients increased significantly after ART as HIV viral load decreased and CD4^+^ T lymphocyte counts increased.^22^ Antiviral drugs also contribute significantly to the increased risk of CVD in HIV patients, and whether early ART ultimately increases or decreases the risk of CVD remains to be clarified by further studies.

HIV infection is an independent risk factor for heart failure, and viral replication is associated with a high risk of heart failure. In a cohort study, involving 2391 and 6095 patients with and without HIV infection, 97 HIV‐infected patients (4.1%) developed heart failure during a mean follow‐up period of 7.3 years.^23^ The most commonly used clinical evaluation markers, LVEF and LVFS, were used to assess cardiac systolic function in our study. The results showed no significant differences in LVEF and LVFS between PLWHA and control groups, nor between AIDS and HIV+ groups, indicating no significant changes in systolic function in PLWHA, consistent with previous studies.^24^, ^25^ Two of the 105 (1.9%) PLWHA in our study had abnormal cardiac systolic function. A Danish study that included 95 consecutive HIV‐infected patients showed that only one case (1%) had a reduced LVEF,^25^ similar to the results of our study. The low incidence of heart failure in our study may be related to the cross‐sectional study design and no long‐term follow‐up.

The results of our study showed that the E/A ratio, lateral e′ velocity, and mitral DT were significantly lower in PLWHA than in healthy controls, indicating that left ventricular diastolic function is reduced in ART‐naïve PLWHA compared to healthy individuals. The main causes of ventricular dysfunction in PLWHA are: direct myocardial damage by HIV, autoimmune damage, damage to the myocardium by increased bioactive substances, and damage to myocardial function due to opportunistic infections.^26^, ^27^ The prevalence of LVDD in PLWHA in our study was 59.0%, which was significantly higher than the 22.5% in the control group. Previous studies have reported a decrease in left ventricular diastolic function in 85.7% of 49 HIV‐infected patients.^28^ The prevalence of LVDD in our study was lower than that in previous studies, which may be related to the fact that all patients in our study were newly diagnosed with HIV infection and had no serious complications. A total of 62 PLWHA suffered from LVDD in our study, most of which (62.9%) had grade 1 diastolic dysfunction, and only 4.8% had grade 3 diastolic dysfunction. A previous prospective study also showed that the majority of HIV‐infected patients with LVDD (35/47, 74.5%) had grade 1 diastolic dysfunction.^24^ Both HIV and the related immune activation and inflammatory responses can increase the risk of abnormal cardiac diastolic function in PLWHA.^29^, ^30^ Cardiac steatosis and myocardial fibrosis may be responsible for the increased incidence of cardiac dysfunction and CVD in PLWHA.^31^, ^32^ Left ventricular structural parameters were not significantly different between AIDS and HIV+ groups. The E/A ratio, a parameter representing diastolic function, was significantly lower in AIDS group than in HIV+ group, and the E/A ratio was positively correlated with CD4 count, which may suggest that the diastolic function of the left ventricle may tend to gradually decrease as the disease progresses in PLWHA.

Risk factors for HIV‐associated CVD include traditional CVD risk factors, ART‐related metabolic disorders, and chronic immune activation and inflammation associated with HIV infection.^33^, ^34^ In the multivariate regression analysis, in addition to the traditional factors of increased age and increased BMI, decreased CD4 cell count was an independent risk factor for LVDD in ART‐naïve PLWHA. CD4 cells are often destroyed in large numbers as target cells for HIV infection, causing severe defects in human immune function. The main change in AIDS is a progressive decrease in CD4 cell and their abnormal function, which ultimately results in a low immune function of the body.^35^ The results of our study showed that the development of cardiac dysfunction in ART‐naïve PLWHA was positively correlated with the severity of CD4 cell damage, and the more severe the immune system damage, the more severe was the cardiac involvement. The lower the number of CD4^+^ cells, the greater the risk of developing LVDD, which is a predictor of further increase in the CVD risk as HIV disease progresses. The median duration of HIV diagnoses in this study was 6 months, and although we cannot currently confirm that the duration of infection is an independent risk factor for LVDD, we found that even limited time living with HIV can impact cardiac structure and function.

## 5 LIMITATION

First, this was a single‐center retrospective study with a relatively small sample size that failed to analyze diastolic dysfunction in PLWHA in a graded and detailed manner. Second, this study used conventional echocardiography, which may have limitations in evaluating the sensitivity of changes in cardiac function and cannot accurately reflect the subtle effects of HIV infection on cardiac function, particularly systolic function. Future studies should be performed with expanded sample size and prospective longitudinal design to further evaluate the changes in cardiac structure and function in PLWHA and their relationship with ART. In addition, in the next study, newer cardiac ultrasound techniques, such as speckle tracking imaging, should be used to accurately assess cardiac function.

## 6 CONCLUSION

ART‐naive PLWHA have a range of structural and functional cardiac changes, with LVDD being a particularly common presentation. Older age, elevated BMI, and decreased CD4 cell counts are independent risk factors for LVDD in ART‐naive PLWHA. To further improve the prognosis of PLWHA, regular echocardiography may be beneficial, particularly in patients with low CD4 cell counts, for early detection of abnormalities, initiation of interventions, control of risk factors, and reduction of CVD risk.

## AUTHOR CONTRIBUTIONS

**Xing Hu**: Conceptualization; writing—original draft; writing—review and editing. **Yuan Zhang**: Formal analysis; writing—original draft. **Tong Zhang**: Conceptualization; writing—review and editing. **Weihua Li**: Methodology; software. **Jing Han**: Methodology. **Xuhui Zhang**: Software. **Fankun Meng**: Conceptualization; writing—review and editing.

## CONFLICT OF INTEREST STATEMENT

The authors declare no conflict of interest.

## ETHICS STATEMENT

The study was approved by the Research Ethics Committee of Beijing Youan Hospital, Capital Medical University (Protocol No. LL‐2020‐084‐K), and informed consent was obtained from all participants.

## Supporting information

Supporting information.

## Data Availability

The datasets generated and analyzed during the current study are not publicly available because they contain information that could compromise the privacy of research participants; however, the data are available from the first author (huxing@ccmu.edu.cn) on reasonable request.
