# Supplementary material for: Echocardiographic assessment of left cardiac structure and function in antiretroviral therapy (ART)‐naïve people living with HIV/AIDS
Source: Immun Inflamm Dis. 2023 Apr 12;11(4):e799. doi: 10.1002/iid3.799 (PMC10091372; doi:10.1002/iid3.799)
Supplement: Supplementary file 1 — Supporting information. [file IID3-11-e799-s001.doc]

**Supplemental Materials**

**Supplementary Table 1 Details of the two patients who had LV systolic dysfunction**

|  |  | Case 1 | Case 2 |
| --- | --- | --- | --- |
| Clinical characteristic | Age (years) | 53 | 37 |
|  | Gender | Male | Male |
|  | CD4+ Count (cells/μL) | 47 | 140 |
|  | Duration of HIV infection (months) | 8 | 6 |
|  | Cardiac symptoms | chest tightness, breathlessness, inability to lie flat at night | chest tightness, choking |
| Cardiac structure | IVST (mm) | 10 | 10 |
|  | LVPWT (mm) | 11 | 10 |
|  | LAD (mm) | 39 | 34 |
|  | LVEDD (mm) | 57 | 50 |
|  | LVESD (mm) | 47 | 38 |
|  | LVMI (g/m^2^) | 119 | 118 |
|  | LAVI (ml/m^2^) | 35 | 32 |
|  | LVEDV (ml) | 158 | 116 |
|  | LVEDI (ml/m^2^) | 75 | 72 |
| Systolic function | LVEF (%) | 37 | 46 |
|  | LVFS (%) | 18 | 23 |
|  | Mitral E/A ratio | 0.57 | 0.89 |
|  | Septal e’ velocity (cm/s) | 10.5 | 7 |
|  | Lateral e’ velocity(cm/s) | 13 | 8 |
|  | Average E/e’ ratio | 4 | 11 |
|  | Mitral DT (ms) | 237 | 127 |
| 10-year risk of CVD |  | 25% | 7% |

1. year risk of CVD was assessed using the Framingham Risk Score (FRS).

IVST: interventricular septum thickness; LVPW: left ventricular posterior wall thickness; LAD: left atrium diameter; LVEDD: left ventricular end-diastolic diameter; LVESD: left ventricular end-systolic diameter; LVMI：left ventricular mass index; LAVI: left atrial volume index; LVEDV: left ventricular end diastolic volume; LVEDI: left ventricular end diastolic volume index; LVEF: left ventricular ejection fraction; LVFS: left ventricular fractional shortening; e': early-diastolic mitral annular velocity; E/A: ratio of early-diastolic LV inflow velocity (E) to atrial-systolic velocity (A); E/e': ratio of early-diastolic LV inflow velocity (E) to early-diastolic mitral annular velocity (e'); DT: deceleration time; CVD: cardiovascular disease

**Supplementary Figure 1 Violin plot showed differences in IVST, IVPWT, LVESD, e’ velocity, and DT between PLWHA and controls, and AIDS and HIV+ groups**


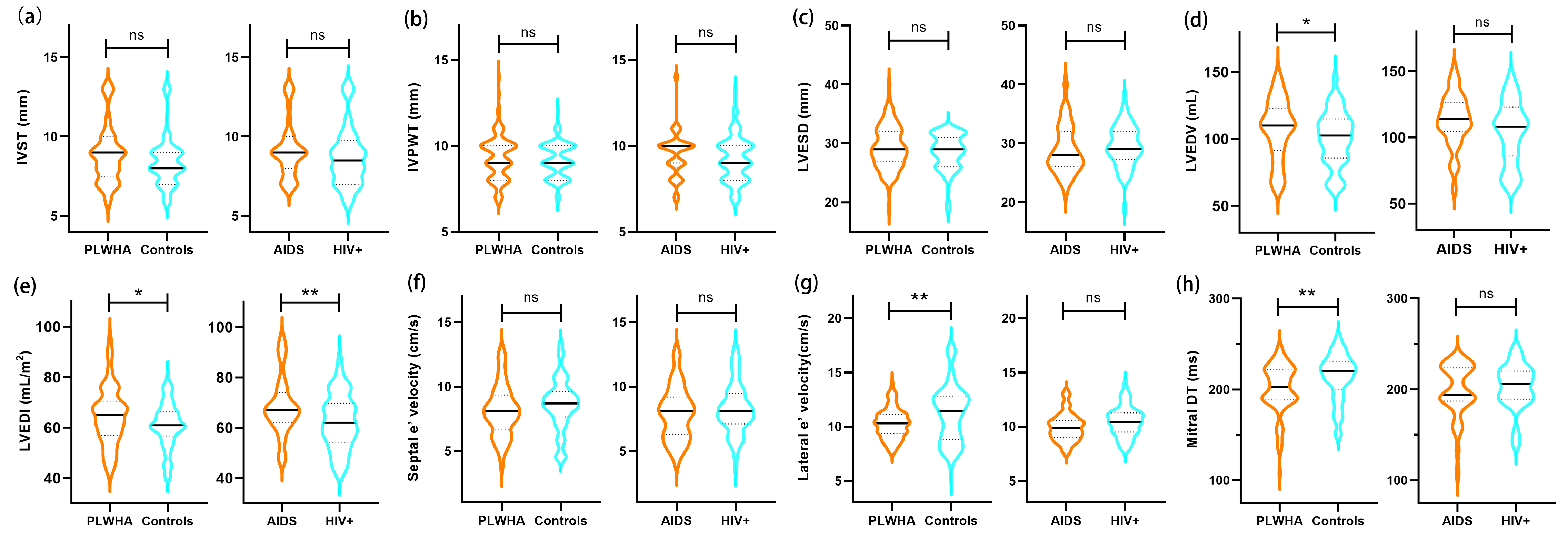


* *p* < 0.05; ns: no significant difference; AIDS: acquired immune deficiency syndrome, HIV: human immunodeficiency virus；IVST: interventricular septum thickness; LVPW: left ventricular posterior wall thickness; LVESD: left ventricular end-systolic diameter; LVEDV: left ventricular end diastolic volume; LVEDI: left ventricular end diastolic volume index; e': early-diastolic mitral annular velocity; DT: deceleration time

**Supplementary Figure 2 Correlation between CD4 cell count and (a) IVST; (b)LVPWT; (c) LAD; (d) LVEDD; (e) LVESD; (f) LVMI; (g) LVAI; (h) LVEDV; (i) LVEDI; (j) Septal e’ velocity; (k) Lateral e’ velocity; (l) Mitral DT in PLWHA**


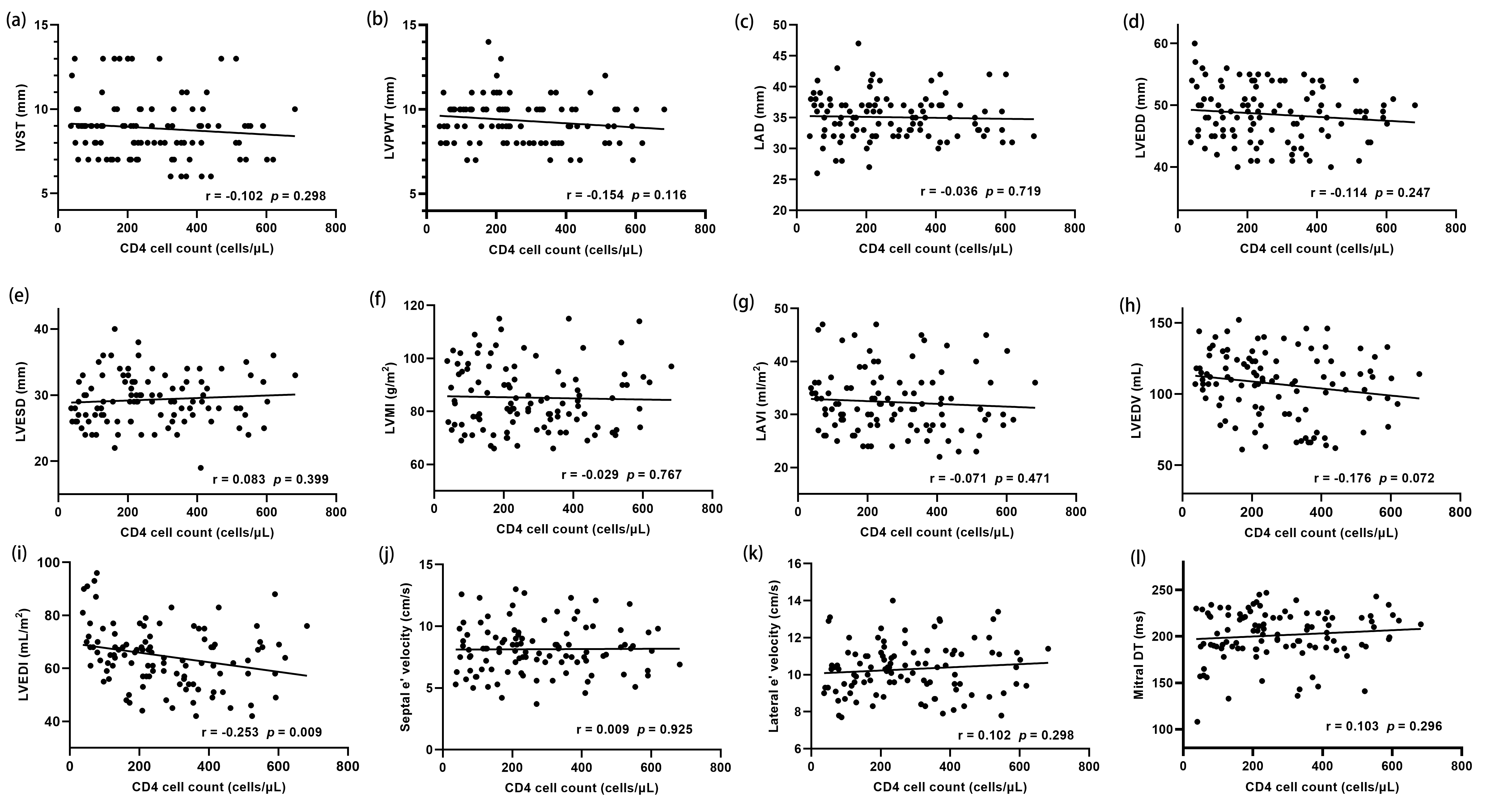


^*^ *p* < 0.05; PLWHA: patients living with HIV/AIDS, IVST: interventricular septum thickness; LVPW: left ventricular posterior wall thickness; LAD: left atrium diameter; LVEDD: left ventricular end-diastolic diameter; LVESD: left ventricular end-systolic diameter; LVMI：left ventricular mass index; LAVI: left atrial volume index; LVEDV: left ventricular end diastolic volume; LVEDI: left ventricular end diastolic volume index; e': early-diastolic mitral annular velocity; DT: deceleration time
